# Supplementary material for: Truly trapped rainbow by utilizing nonreciprocal waveguides
Source: Sci Rep. 2016 Jul 25;6:30206. doi: 10.1038/srep30206 (PMC4958975; doi:10.1038/srep30206)
Supplement: Supplementary Information [file srep30206-s1.pdf]

Supplementary Information

Truly trapped rainbow by utilizing nonreciprocal waveguides

Kexin Liu[1,2] and Sailing He[1,2,*]

1. Department of Electromagnetic Engineering, School of Electrical Engineering, KTH Royal Institute of Technology, Stockholm S-100 44, Sweden

2. Centre for Optical and Electromagnetic Research, Zhejiang Provincial Key Laboratory for Sensing Technologies, JORCEP (Sino-Swedish Joint Research Center of Photonics), Zhejiang University, Hangzhou 310058, China

**Corresponding author: sailing@kth.se*

A. Method for time domain simulation

The method for time domain simulation is the same as the method in [1]. In our paper, the propagation of the wave packet in time domain is formed by the superposition of 51 modulated frequency components with a central frequency $\omega_c = 1.1\omega_m$. Each frequency component $\psi(\omega_n)$ is obtained by a frequency domain simulation with exciting a 1A line current at $x = -0.5\lambda_m$ in COMSOL. The frequency sequence ω_n is an arithmetic sequence from $1.08\omega_\mathrm{m}$ to $1.12\omega_\mathrm{m}$ with common difference of $\Delta\omega = \omega_m/1250$. The amplitude of each component is modulated by a Gaussian function $A(\omega_n) = \exp[-\frac{1}{200}\left(\frac{\omega_n-\omega_c}{\Delta\omega}\right)^2]$ as shown in Fig. S1. The superposition of the modulated components gives the propagation of the wave packet Ψ in time domain by the following formula

$$\Psi = \sum_n A(\omega_n)\,\psi(\omega_n)\exp(i\omega_n t) \tag{S1}$$

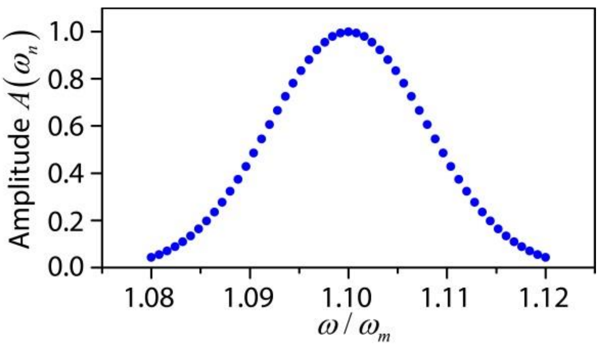

Figure S1 | Gaussian function $A(\omega_n)$. 51 frequency components are modulated by the Gaussian function $A(\omega_n)$ to construct the propagation of the wave packet in time domain.

B. Reflection caused by disorders in reciprocal waveguides

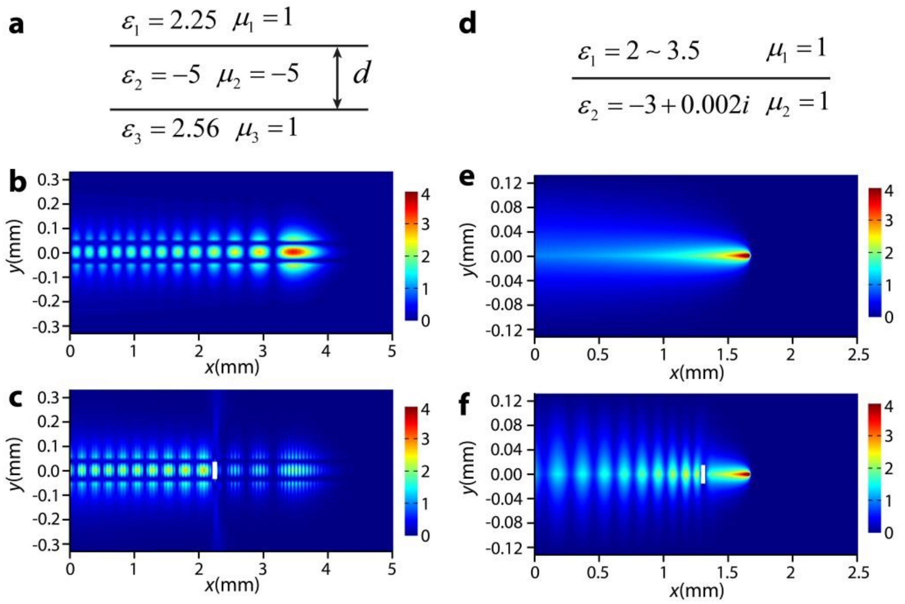

Figure S2 | Reflection caused by disorders in reciprocal waveguides. a, Structure of a three layer tapered waveguide. The bottom and upper layers are dielectric materials with $\varepsilon_1 = 2.25$, $\mu_1 = 1$ and $\varepsilon_3 = 2.56$, $\mu_3 = 1$. The middle layer is double negative metamaterial with $\varepsilon_2 = -5$ and $\mu_2 = -5$. The thickness d of the middle layer is changing linearly from 55um to 51.5um with x from 0mm to 5mm. The frequency is 1THz and the critical thickness is 52.45um. **b,** Distribution of $|H_z|$ without disorders for the waveguide in **a**. **c,** Distribution of $|H_z|$ with a dielectric slab (white rectangle) with $\varepsilon_r = 10$, $\mu_r = 1$ and $\Delta x = \Delta y = 0.7$mm inserted in the waveguide in **a**. **d,** Structure of a two layer waveguide. The upper layer is a dielectric layer with ε_1 changing from 2 to 3.5 with x from 0mm to 2.5mm. The bottom layer has negative permittivity $\varepsilon_2 = -3 + 0.002i$. The operation frequency is 0.3GHz and the critical value of ε_1 is -3. **e,** Distribution of $|H_z|$ without disorders for the waveguide in **d**. **f,** Distribution of $|H_z|$ with a dielectric slab (white rectangle) with $\varepsilon_r = 10$, $\mu_r = 1$ and $\Delta x = \Delta y = 0.03$mm inserted in the waveguide in **d**.

Compared with the nonreciprocal waveguides suggested in our paper, the reciprocal waveguides are sensitive to the disorders. We study two slow wave structures to show the influence of the disorders. The first one is a three layer tapered waveguide with the same material parameters in [1, 2] (Fig. S2a). The bottom and upper layers are dielectric materials with $\varepsilon_1 = 2.25$, $\mu_1 = 1$ and $\varepsilon_3 = 2.56$, $\mu_3 = 1$. The middle layer is double negative metamaterial with $\varepsilon_2 = -5$ and $\mu_2 = -5$. The thickness d of the middle layer is changing linearly from 55um to 51.5um with x from 0mm to 5mm. The frequency is 1THz and the critical thickness is 52.45um where the group velocity v_g is zero. Figure S2b shows the distribution of $|H_z|$ without disorders. Figure S2c shows the distribution of $|H_z|$, when we insert a dielectric slab (white rectangle) with $\varepsilon_r = 10$, $\mu_r = 1$ and $\Delta x = \Delta y = 0.7$mm. The disorder generates direct reflection in this waveguide. The second one is a two layer structure

supporting surface wave (Fig. S2d). The upper layer is a dielectric layer with ε_1 changing from 2 to 3.5 with x from 0mm to 2.5mm. The bottom layer has negative permittivity $\varepsilon_2 = -3 + 0.002i$. The operation frequency is 0.3GHz and the critical value of ε_1 is -3 where $v_g \to 0$ and the wave vector $k \to \infty$. Figure S2e shows the distribution of $|H_z|$ without disorders. Figure S2f shows the distribution of $|H_z|$, when we insert a dielectric slab (white rectangle) with $\varepsilon_r = 10$, $\mu_r = 1$ and $\Delta x = \Delta y = 0.03$mm. The disorder can also generate direct reflection in this waveguide.

C. Information about supplementary movies

(1) Supplementary Movie 1

Propagation of a Gaussian wave packet in the waveguide with $d = 0.13\lambda_m$. The center frequency is $\omega_c = 1.1\omega_m$ and the evolution of the E_z amplitude distribution is recorded in the movie.

(2) Supplementary Movie 2

Propagation of a Gaussian wave packet in the waveguide with $d = 0.013\lambda_m$. The center frequency is $\omega_c = 1.1\omega_m$ and the evolution of the E_z amplitude distribution is recorded in the movie.

References

1. He, S., He, Y. & Jin, Y. Revealing the truth about 'trapped rainbow' storage of light in metamaterials. *Sci. Rep.* **2**, 583 (2012).
2. Tsakmakidis, K. L., Boardman, A. D. & Hess, O. 'Trapped rainbow' storage of light in metamaterials. *Nature* **450**, 397-401 (2007).
